# Assessment of self-health management capacity and its influencing factors in patients with decompensated liver cirrhosis in Shanxi Province, China

**DOI:** 10.3389/fpubh.2025.1662613

**Published:** 2025-11-10

**Authors:** Feixue Li, Jing Wang, Hongping Wen

**Affiliations:** Department of Pharmacy, Shanxi Provincial People's Hospital, Taiyuan, China

**Keywords:** decompensated liver cirrhosis, self-health management capacity, influencing factors, Shanxi Province, intervention strategy

## Abstract

**Objective:**

This research aimed to evaluate the current status of self-health management capacity among patients with decompensated hepatic cirrhosis in Shanxi Province, China, and to analyze its influencing factors.

**Methods:**

A questionnaire survey was performed from October 2024 to February 2025 at Shanxi Provincial People’s Hospital, targeting patients with decompensated liver cirrhosis. Self-developed questionnaires were employed to measure self-health management capacity and identify associated influencing factors.

**Results:**

A total of 460 valid responses were collected. The scale demonstrated excellent internal consistency reliability, with a Cronbach’s alpha of 0.94, and each dimension’s Cronbach’s alpha ranged from 0.78 to 0.90. The overall construct validity, indicated by the KMO was 0.93, with individual dimension KMO values between 0.68 and 0.88. The average score of self-health management ability was 3.04 ± 0.51 out of 5. Correlation analysis showed that cognitive ability, psychological status, behavioral lifestyle, and treatment adherence had very strong positive correlations with overall self-management capacity (0.8 < *r* ≤ 1, *p* < 0.001). The multiple linear regression model yielded an *R*-squared value of 0.687 (*p* < 0.001). Multivariate analysis indicated that higher educational attainment, increased household income, and longer disease duration were significantly positively associated with self-management scores (positive Beta coefficients, *p* < 0.05). Conversely, occupational status, healthcare burden, alcohol consumption history, comorbidities, and Child-Pugh classification were significantly negatively associated with self-management capacity (negative Beta coefficients, *p* < 0.05).

**Conclusion:**

The self-health management capacity among patients with decompensated liver cirrhosis in Shanxi Province is moderate to low, influenced by various determinants such as education level, occupational status, monthly household income, healthcare burden, duration of liver cirrhosis, alcohol consumption history, comorbidities, and Child-Pugh classification. It is recommended a precise intervention strategy is proposed to enhance the public health prevention and control effect of liver cirrhosis: transitioning from traditional hospital-centric models to a stratified, intelligent, and community-integrated comprehensive prevention and control system.

## 1 Introduction

Cirrhosis is a pathological stage resulting from the progression of chronic liver disease, characterized by diffuse hepatic fibrosis, pseudolobule formation, and proliferation of intrahepatic and extrahepatic vasculature. Patients in the compensated phase often exhibit no significant clinical symptoms, whereas decompensated cirrhosis is marked by portal hypertension and severe hepatic functional impairment. Complications such as ascites, gastrointestinal hemorrhage, sepsis, hepatic encephalopathy, hepatorenal syndrome, or malignant transformation frequently lead to multi-organ failure and mortality (1, 2). Notably, the unplanned readmission rate within 1 month post-discharge for decompensated liver cirrhosis (DLC) patients can reach approximately 26% (3), which significantly impacts quality of life and imposes substantial economic burdens on families and society, representing a major global public health challenge (4–7). In the context of limited healthcare resources, enhancing patients’ self-management of health is of urgent practical importance to reduce unplanned readmissions and optimize resource allocation (8–10).

However, the self-management status among patients with DLC in our country is concerning (11). Research by Zhu et al. (12) indicates that only 6.8% of patients demonstrate effective daily management. Interviews conducted by Li et al. (13) reveal prevalent issues with medication adherence, including a lack of understanding of proper pharmacological use and self-discontinuation due to concerns about adverse drug reactions. Additionally, there is a significant demand for scientifically guided dietary management (14–16). Therefore, it is crucial for healthcare professionals to investigate the factors influencing this population’s health self-management capabilities and to optimize intervention strategies accordingly to enhance their self-care levels (17–22).

In recent years, the field of chronic disease self-management research within the domestic healthcare sector has experienced rapid development, particularly concerning conditions such as hypertension (23, 24), diabetes (25–27), coronary artery disease (28, 29) chronic obstructive pulmonary disease (30), and fatty liver (31). However, the majority of studies focus on isolated aspects such as nutrition, diet, medication adherence, or psychological counseling. In contrast, research specifically targeting liver cirrhosis remains limited in scale and often fails to distinguish between compensated and decompensated stages (32–36). Given the significant differences in physical and mental health status between these stages, it is crucial to investigate whether self-management capabilities and influencing factors are consistent across stages.

**Figure 1:**
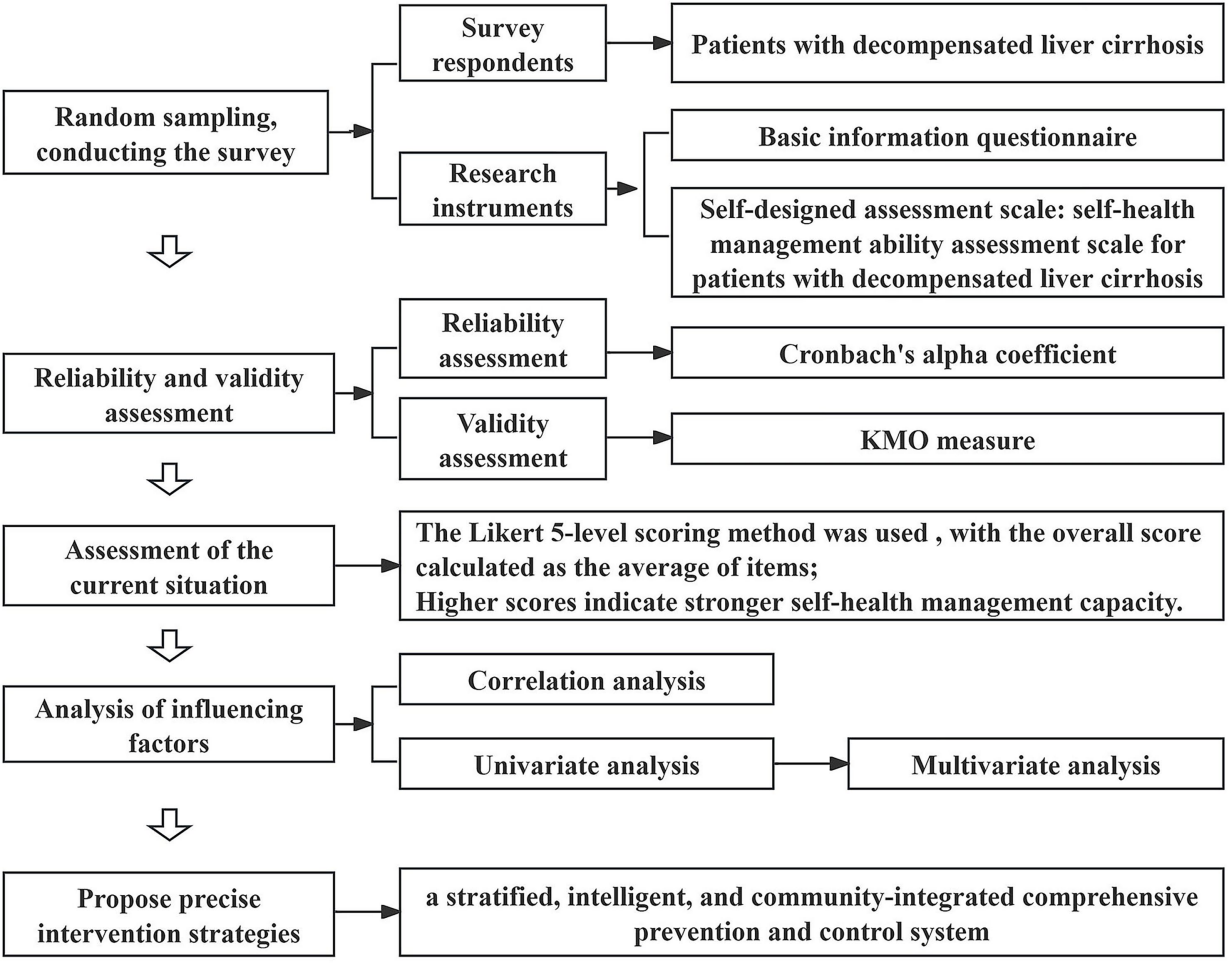
A schematic diagram in this research.

To address this, this study employs a self-developed “Self-Health Management Ability Assessment Scale for Patients with DLC” to evaluate patients with DLC admitted to Shanxi Provincial People’s Hospital in Shanxi Province, China. The objectives are to validate the scale’s feasibility, identify key determinants affecting patients’ self-health management capacity, and propose targeted intervention strategies based on the findings, thereby providing evidence for personalized health management and precise clinical interventions. A schematic diagram of the research is shown in Figure 1.

## 2 Research subjects and methods

### 2.1 Research subjects

#### 2.1.1 Selection criteria for participants

A convenience sampling technique was employed to select hospitalized patients diagnosed with DLC at Shanxi Provincial People’s Hospital from October 2024 to February 2025. Participants were administered a self-developed “Self-Health Management Ability Assessment Scale for Patients with DLC” and their responses were collected.

#### 2.1.2 Inclusion and exclusion criteria

##### 2.1.2.1 Inclusion criteria

(1) Diagnosis of DLC according to the clinical guidelines (1) for cirrhosis management;
(2) Age ≥18 years;
(3) Awareness of their condition and voluntary informed consent;
(4) No prior participation in psychological surveys or intervention studies.

##### 2.1.2.2 Exclusion criteria

(1) Presence of severe dysfunction in cardiac, cerebral, renal, or other vital organs;
(2) Complete loss of self-care ability.

#### 2.1.3 Sample size calculation

Based on the principle that the sample size for questionnaire surveys should be 5–10 times the number of variables (with the current study having 39 variables), the initial sample size was estimated at 390 cases using the maximum multiplier (10 times). Considering a 10% invalid questionnaire rate, the final sample size was determined to be 429 cases. A total of 500 questionnaires were distributed, with 460 valid responses collected, resulting in a valid response rate of 92%. All the retrieved questionnaires were checked for completeness to ensure no missing items. The incomplete questionnaires were excluded.

### 2.2 Research methods

An electronic questionnaire survey was conducted via “Questionnaire Star.” The questionnaire was designed using the Delphi expert consultation method (37), revised by specialists in the relevant field, to collect demographic data and clinical information related to disease diagnosis and treatment. This study received approval from the Ethical Committee of Shanxi Provincial People’s Hospital in Shanxi Province, China (No. 804 in 2024).

#### 2.2.1 Data collection methods

(1) Uniform training of investigators: two clinical pharmacists with the title of associate chief pharmacists from the Pharmacy Department of Shanxi Provincial People’s Hospital served as experts to provide guidance. Through the training, each investigator was required to clearly understand the content of the questionnaire, the purpose of the investigation, master the methods of questionnaire inquiries, filling out forms, as well as the possible problems and corresponding solutions during the investigation, to ensure the consistency of the investigation;
(2) Informed consent: explaining the study significance to eligible patients and obtaining consent before proceeding;
(3) Standardized guidance: providing uniform instructions to thoroughly explain questionnaire requirements;
(4) Assistance with completion: for individuals with reading difficulties, investigators read and explained each item aloud and completed the questionnaire on their behalf according to their wishes.

#### 2.2.2 Research instruments

(1) Basic Information Questionnaire: covering gender, age, marital status, number of children, residential situation, place of residence, education level, occupational status, monthly household income, medical expense payment methods, healthcare burden, type of liver cirrhosis, use of anti-viral therapy, use of anti-fibrotic therapy, frequency of complications within 3 months, duration of liver cirrhosis, family history of liver cirrhosis, smoking history, alcohol consumption history, comorbidities, and Child-Pugh classification.
(2) Self-Designed Assessment Scale: the “Self-Health Management Ability Assessment Scale for Patients with DLC” encompasses five dimensions—cognitive ability, psychological status, behavioral lifestyle, social environment and treatment adherence—with a total of 39 items. The Likert 5-level scoring method was used (1 = “Never” to 5 = “Always”), with the overall score calculated as the average of items; higher scores indicate stronger self-health management capacity.

### 2.3 Statistical methods

Data exported from “Questionnaire Star” were analyzed using SPSS 25.0. Quantitative data conforming to a normal distribution are expressed as $\bar{x}\pm s$ , while qualitative data are described using frequencies and percentages (%). Single-factor analysis utilized *t*-tests (binary variables) or ANOVA (multicategory variables); multivariate analysis employed multiple linear regression. *p* < 0.05 was considered statistically significant.

## 3 Results

### 3.1 Reliability and validity assessment of the scale

This study is based on the theories of social cognition, self-efficacy, self-determination, disease risk factor accumulation, and health ecology model, laying a theoretical foundation for the construction of the self-management ability assessment system for patients with decompensated liver cirrhosis. During the scale development stage, based on literature review and Delphi expert consultation (with two rounds of evaluation by 15 experts), an assessment index system for the self-health management ability of patients with decompensated liver cirrhosis was constructed on the basis of the “Self-Management Behavior Scale for Patients with Liver Cirrhosis” compiled by Wang et al. (33) Given that the scale is based on expert consensus, it possesses strong content validity. Consequently, this research focused solely on assessing internal consistency reliability (using Cronbach’s alpha coefficient) and structural validity (using the KMO measure).

The reliability analysis results indicate that the overall scale’s Cronbach’s alpha coefficient is 0.94; the Cronbach’s alpha coefficients for the primary dimensions range from 0.78 to 0.90 (Table 1), demonstrating strong internal consistency for the entire scale and its subdomains.

**Table 1:** Reliability and validity assessment of the scale (*n* = 460).

	Cronbach’s alpha coefficient (*α*)	KMO
Cognitive abilities (Items 1–5)	0.90	0.79
Psychological status (Items 6–8)	0.85	0.72
Behavioral lifestyle (Items 9–23)	0.86	0.88
Social environment (Items 24–26)	0.78	0.68
Treatment adherence (Items 27–39)	0.87	0.87
Overall scale (Items 1–39)	0.94	0.93

The construct validity assessment shows a KMO measure of 0.93 for the total scale; the KMO values for individual primary dimensions range from 0.68 to 0.88 (Table 1), indicating that the scale is suitable for factor analysis.

### 3.2 Scores across various dimensions of self-health management capacity in patients with DLC

The survey results (Table 2) indicate that the overall self-management ability score among patients with DLC in Shanxi Province is 3.04 ± 0.51 out of 5. The domain scores, ranked from highest to lowest, are as follows: social environment (3.42 ± 0.56), treatment adherence (3.23 ± 0.56), behavioral lifestyle (3.14 ± 0.52), psychological status (2.69 ± 0.79), and cognitive ability (2.17 ± 0.74).

**Table 2:** Total score and dimension scores of the self-health management ability scale for patients with DLC (*n* = 460).

	Score ( $\bar{x}\pm s$ )
Cognitive abilities (Items 1–5)	2.17 ± 0.74
Psychological status (Items 6–8)	2.69 ± 0.79
Behavioral lifestyle (Items 9–23)	3.14 ± 0.52
Social environment (Items 24–26)	3.42 ± 0.56
Treatment adherence (Items 27–39)	3.23 ± 0.56
Overall scale (Items 1–39)	3.04 ± 0.51

### 3.3 Analysis of factors influencing self-health management capacity in patients with DLC

#### 3.3.1 Correlation analysis of factors affecting self-health management capacity in patients with DLC

Table 3 demonstrates significant positive correlations among five domains—cognitive ability, psychological status, behavioral lifestyle, social environment, and treatment adherence—and their association with overall self-health management capacity in patients (*p* < 0.001).

(1) Correlation with overall capacity: cognitive ability, psychological status, behavioral lifestyle, and treatment adherence exhibit very strong positive correlations with total self-health management capacity (0.8 < *r* ≤ 1); the social environment domain shows a strong positive correlation (0.6 < *r* ≤ 0.8).
(2) Inter-domain correlations: cognitive ability correlates strongly with psychological status, behavioral lifestyle, and treatment adherence (0.6 < *r* ≤ 0.8). Psychological status correlates strongly with behavioral lifestyle, social environment, and treatment adherence (0.6 < *r* ≤ 0.8). Behavioral lifestyle correlates strongly with social environment and treatment adherence (0.6 < *r* ≤ 0.8).

**Table 3:** Pearson correlation matrix of self-health management competence in patients with DLC (*n* = 460).

	Cognitive ability	Psychological status	Behavioral lifestyle	Social environment	Treatment adherence	Overall score
Cognitive ability	1.000	0.777^**^	0.687^**^	0.561^**^	0.689^**^	0.856^**^
Psychological status		1.000	0.683^**^	0.621^**^	0.628^**^	0.823^**^
Behavioral lifestyle			1.000	0.611^**^	0.648^**^	0.899^**^
Social environment				1.000	0.518^**^	0.699^**^
Treatment adherence					1.000	0.876^**^
Overall score						1.000

^**^Indicates *p* < 0.001.

These findings indicate that each domain and their interrelationships significantly influence patients’ overall self-health management capacity, providing a scientific basis for developing targeted and effective health intervention strategies.

#### 3.3.2 Univariate analysis of factors influencing self-health management capacity in patients with DLC

When the dependent variable is continuous, linear regression analysis is typically employed to examine the association between independent and dependent variables. Patient characteristics and univariate analysis of self-health management ability in DLC are presented in Table 4. Among them, 263 cases were male (57.2%), and 197 cases were female (42.8%). The findings indicate that self-management levels are significantly correlated with age, marital status, number of children, residential situation, place of residence, educational level, occupational status, monthly household income, medical expense payment method, healthcare burden, whether anti-fibrotic therapy is administered, frequency of complications within 3 months, duration of liver cirrhosis, smoking history, alcohol consumption history, comorbidities, and Child-Pugh classification (*p* < 0.05). The variance inflation factor (VIF) values for all variables were all below 5 (ranging from 1.048 to 4.317), suggesting the absence of severe multicollinearity issues.

**Table 4:** Patient characteristics and univariate analysis of self-health management ability in DLC (*n* = 460).

Factors	Variable assignment	Number of participants	Composition ratio (%)	*t/F*	*p*	VIF
Gender	Female = 0	197	42.82	−0.746	0.456	2.127
	Male = 1	263	57.17			
Age (years)	Under 40 = 0	30	6.52	2.487	0.031	2.471
	41–50 = 1	84	18.26			
	51–60 = 2	140	30.43			
	61–70 = 3	123	26.74			
	Over 70 = 4	83	18.04			
Marital status	Unmarried = 0	9	1.96	5.594	0.001	1.377
	Married = 1	405	88.04			
	Divorced = 2	5	1.09			
	Widowed = 3	41	8.91			
Number of children	None = 0	14	3.04	19.927	0.000	1.521
	One = 1	130	28.26			
	Two = 2	191	41.52			
	Three = 3	90	19.57			
	More than three = 4	35	7.61			
Residential situation	Living with spouse = 0	353	76.74	4.275	0.005	1.417
	Living with children = 1	87	18.91			
	Living alone = 2	20	4.35			
Place of residence	Rural = 0	78	16.96	56.201	0.000	1.778
	Township = 1	138	30.00			
	Urban = 2	244	53.04			
Educational level	Primary school or below = 0	167	36.30	141.405	0.000	4.317
	Junior high school = 1	132	28.70			
	Senior high school or vocational school = 2	116	25.22			
	University or above = 3	45	9.78			
Occupational status	Retired = 0	103	22.39	82.750	0.000	2.473
	Employed = 1	200	43.48			
	Unemployed = 2	157	34.13			
Monthly household income (RMB)	<3,000 = 0	122	26.52	150.027	0.000	3.956
	3,000–4,999 = 1	104	22.61			
	5,000–10,000 = 2	218	47.39			
	>10,000 = 3	16	3.48			
Medical expense payment method	Resident medical insurance = 0	289	62.83	−15.930	0.000	3.510
	Employee medical insurance = 1	171	37.17			
Healthcare burden	No burden = 0	26	5.65	172.908	0.000	3.484
	minimal burden = 1	222	48.26			
	Moderate burden = 2	175	38.04			
	high burden = 3	37	8.04			
Type of liver cirrhosis	Post-hepatitis cirrhosis = 0	172	37.39	2.174	0.056	2.271
	Primary biliary cirrhosis = 1	122	26.52			
	alcoholic cirrhosis = 2	75	16.30			
	Other causes of cirrhosis (e.g., drug-induced) = 3	2	0.43			
	Unknown = 4	89	19.35			
Use of anti-viral therapy	Never received = 0	290	63.04	1.490	0.226	2.448
	Previously received = 1	7	1.52			
	Currently undergoing treatment = 2	163	35.43			
Use of anti-fibrotic therapy	Never received = 0	90	19.57	5.371	0.005	1.171
	Previously received = 1	3	0.65			
	Currently undergoing treatment = 2	367	79.78			
Frequency of complications within 3 months	Once = 0	353	76.74	3.119	0.045	1.143
	Twice = 1	86	18.70			
	More than three times = 2	21	4.57			
Primary complication type leading to this hospitalization	Esophagogastric variceal bleeding = 0	310	67.39	0.565	0.784	1.048
	Ascites = 1	53	11.52			
	Hypersplenism = 2	26	5.65			
	Hepatic encephalopathy = 3	32	6.96			
	Acute and chronic liver failure = 4	20	4.35			
	Primary liver cancer = 5	14	3.04			
	Hepatorenal syndrome = 6	5	1.09			
Duration of liver cirrhosis	Less than 1 year = 0	114	24.78	2.717	0.044	1.757
	1–5 years = 1	137	29.78			
	5–10 years = 2	103	22.39			
	over 10 years = 3	106	23.04			
Previous hospitalizations due to liver cirrhosis	Once = 0	36	7.83	2.990	0.051	1.729
	2–3 times = 1	84	18.26			
	More than 3 times = 2	340	73.91			
Family history of liver disease	No = 0	409	88.91	0.516	0.606	1.192
	Yes = 1	51	11.09			
Smoking history	Never smoked = 0	319	69.35	3.733	0.025	2.479
	Former smoker (quit) = 1	81	17.61			
	Current smoker = 2	60	13.04			
Alcohol consumption history	Never drank = 0	334	72.61	7.456	0.001	2.676
	Former drinker (quit) = 1	107	23.26			
	Current drinker = 2	19	4.13			
Comorbidities	No = 0	240	52.17	2.699	0.007	1.140
	Yes = 1	220	47.83			
Child-Pugh classification	Grade A = 0	3	0.65	43.000	0.000	1.207
	Grade B = 1	350	76.09			
	Grade C = 2	107	23.26			

#### 3.3.3 Multivariate analysis of factors influencing self-health management capacity in DLC patients

Initially, potential relevant predictors were preliminarily identified based on univariate analysis results (*p* < 0.05). Subsequently, all candidate variables underwent multicollinearity assessment through VIF (VIF < 5). Furthermore, based on literature (17), we removed five variables considered less relevant to self-management ability (e.g., age and number of children). Eventually, 12 key variables were retained for the multivariate linear regression analysis to explore the determinants affecting self-management levels in DLC patients (Table 5).

**Table 5:** Multivariate linear regression analysis of factors affecting self-health management capacity in patients with DLC (*n* = 460).

Dependent variable	Independent variable	Beta	*t*	*p*	VIF	*R*^2^	*F*	*p*
Self-health management ability score of patients with DLC	Marital status	−0.041	−1.517	0.130	1.083	0.688	85.315	0.000
	Educational level	0.357	8.906	0.000	2.365			
	Occupational status	−0.106	−3.792	0.000	1.147			
	Monthly household income	0.246	4.912	0.000	3.698			
	Healthcare burden	−0.185	−3.990	0.000	3.173			
	Use of anti-fibrotic therapy	0.037	1.383	0.167	1.061			
	Frequency of complications within 3 months	0.003	0.102	0.919	1.068			
	Duration of liver cirrhosis	0.063	2.344	0.020	1.056			
	Smoking history	−0.034	−0.898	0.370	2.157			
	Alcohol consumption history	−0.174	−4.414	0.000	2.285			
	Comorbidities	−0.073	−2.709	0.007	1.074			
	Child-Pugh classification	−0.169	−5.921	0.000	1.192			

The model’s coefficient of determination (*R*^2^) was 0.688, indicating a high degree of fit, with the selected predictors accounting for 68.8% of the variance in self-management scores. The *F* was 85.315 with *p* < 0.001, demonstrating the overall statistical significance of the model, whereby variations in independent variables significantly influence patients’ self-management scores.

Results from the multivariate regression indicate that educational level, occupational status, monthly household income, healthcare burden, duration of liver cirrhosis, alcohol consumption history, comorbidities, and Child-Pugh classification are significant factors affecting self-health management capacity in DLC patients in Shanxi Province (*p* < 0.05). Specifically, higher educational levels, greater household income, and longer disease duration are positively correlated with self-management scores (positive Beta coefficients). Conversely, occupational status, healthcare burden, alcohol use history, comorbidities, and Child-Pugh grades are negatively correlated with self-health management capacity (negative Beta coefficients).

## 4 Discussion

### 4.1 Current status of self-health management ability of patients with DLC

In this study, the self-developed “Self-Health Management Ability Assessment Scale for Patients with DLC” was used to conduct on-site evaluation of patients with DLC who were hospitalized in Shanxi Provincial People’s Hospital in Shanxi Province, China, from October 2024 to February 2025. The results showed that the average score of patients’ self-health management ability was 3.04 ± 0.51, which was at the lower middle level overall. In the dimensions of “social environment,” “treatment compliance” and “behavior and lifestyle,” the patients performed relatively well, indicating that they were able to obtain effective family support, follow the doctor’s instructions, take medication in accordance with the prescribed schedule, dosage, and treatment course, and seek medical attention in time when symptoms such as gastric discomfort, hematemesis, progressive increase in abdominal circumference or edema of the lower limbs occurred, and pay attention to daily diet and lifestyle management. However, the low scores of patients in the dimensions of “cognitive ability” and “psychological status” suggest that they have insufficient opportunities to participate in health education, and there is a common psychological stress and cognitive impairment related to the disease.

### 4.2 Correlation and key influencing factors of self-health management ability of patients with DLC

Based on the results of the correlation analysis, “behavioral lifestyle” and “treatment compliance” showed the most significant correlation with the total score of patients’ self-health management ability, clearly indicating that these two factors are the core controllable targets influencing the self-management efficacy of patients with DLC. This finding provides a crucial empirical basis for the subsequent construction of precise and structured intervention strategies.

The self-health management ability of patients with DLC is affected by multiple factors. Education level, occupational status, monthly family income, healthcare burden, duration of liver cirrhosis, alcohol consumption history, comorbidities, and Child-Pugh classification were key influencing factors. The research findings align with existing scholarly literature (17–21) and provide a theoretical basis for the formulation of precise and stratified intervention strategies. The analysis of the influencing factors is as follows:

(1) Education level: patients with higher education level showed better self-health management ability. This is closely related to its strong ability to acquire health knowledge, understand and actively learn, so that it can manage health more effectively. Patients with a low level of education are relatively under-educated in this regard. Although it is a long-term goal to improve the cultural literacy of the whole people, it is possible to improve the health concept of patients with DLC and improve their management ability by strengthening targeted health education and knowledge popularization.
(2) Occupational status: occupational status has a significant impact on self-health management ability. The ability of retired patients to manage their own health is relatively high, while that of unemployed patients is lower. The reason may be that retired patients usually have a relatively high education level and income level, and are more willing to invest time and energy in learning about liver cirrhosis, and have a certain understanding of complication prevention and drug effects. Unemployed patients often have a higher incidence of complications and limited management ability due to low education level, lack of disease knowledge, and insufficient awareness of complication prevention. In addition, financial pressure may prompt unemployed patients to stop taking their medication without medical advice or to believe in unproven remedies, which seriously affects the treatment outcome and health.
(3) Monthly household income: the monthly household income was positively correlated with self-health management ability. Higher incomes provide economic guarantee for patients and make them more able to follow up with treatment requirements, such as regular follow-ups, adherence to doctor’s orders, and standardized medication, and avoid discontinuation or abandonment of treatment due to financial pressure.
(4) Healthcare burden: the heavier the healthcare burden, the lower the patient’s ability to manage their own health. Heavy financial pressures not only directly affect patients, but can also weaken family members’ support for their treatment. Therefore, medical staff should focus on low-income and unemployed patient groups with heavy healthcare burden, strengthen health education, and reduce the frequency of hospitalization due to complications by improving their self-health management level, so as to avoid further aggravation of medical burden.
(5) Duration of liver cirrhosis: with the prolongation of the course of the disease, the patient’s self-health management ability has been improved. The long-term course of the disease provides patients with more opportunities to receive health guidance from medical professionals, accumulate disease-related knowledge, and deepen their understanding of the importance of self-health management. It is suggested that health education and medication guidance for patients with liver cirrhosis should be carried out in a planned and phased manner, relevant knowledge should be popularized, a scientific and healthy lifestyle should be advocated, and the positive role of health education should be fully played.
(6) Alcohol consumption history: alcohol consumption history is a key factor affecting patients’ self-health management ability. The liver function of patients with DLC has been severely impaired, and long-term heavy alcohol consumption will further increase the burden on the liver, resulting in poor health management effect. Therefore, it must be emphasized that patients with cirrhosis should strictly follow the doctor’s instructions to abstain from alcohol in order to improve the quality of life.
(7) Comorbidities: the higher the number of comorbidities, the lower the level of self-health management of patients. The increase in comorbidities leads to more complex medication regimens, overlapping symptoms, and an increase in the amount of health and disease knowledge that patients need to master. This, in turn, raises the requirements for their self-management ability. The two factors interact with each other, easily leading to a vicious cycle of poor management results. It is suggested to implement individualized health follow-up, and carry out targeted health education and supervision based on the specific conditions of the patients, in order to improve their self-management level.
(6) Child-Pugh classification: the Child-Pugh classification is an important clinical indicator to evaluate the severity of liver reserve function in patients with liver cirrhosis (grade A is the mildest, grade C is the heaviest). The higher the grade (i.e., the worse the liver function), the lower the patient’s level of self-management. This may be due to the fact that exacerbations are accompanied by multiple complications that increase the need for self-management; At the same time, decompensated symptoms such as abdominal distension, fatigue, and sleep disturbance consume patients’ energy and weaken their management ability. Therefore, on the basis of fully understanding their own health status, patients should have regular physical examinations to assess their conditions, strictly follow the doctor’s instructions and timely adjust the treatment plan. At the same time, they should make scientific adjustments to their diet structure, behavioral lifestyle, and exercise regimen, establish and follow an effective self-management plan, in order to reduce complications and improve the quality of life.

### 4.3 Prospects and limitations

Based on the results of correlation analysis and key influencing factors, we propose targeted precision intervention strategies to enhance the self-health management ability of patients with DLC in Shanxi Province. This involves transitioning from traditional hospital-centric models to a stratified, intelligent, and community-integrated comprehensive prevention and control system. Specific measures include: (1) leveraging the AI-powered digital pharmacist services implemented by the Pharmacy Department of Shanxi Provincial People’s Hospital—covering intelligent medication management and personalized pharmaceutical consultations—to promote the downward transfer of digital resources from provincial hospitals to grassroots levels, and achieve intelligent medication warning and systematic long-term follow-up for high-risk patients; (2) establish a stratified health education system based on educational level and clinical stage, utilize resources such as health science popularization videos released by the WeChat official account of Shanxi Provincial People’s Hospital, to enhance health education for people with low educational levels. The core lies in extending the multidisciplinary collaboration (MDT) model to the community, using co-morbidity management as the entry point, integrating resources from multiple disciplines such as liver diseases, cardiovascular diseases, endocrinology, and pharmacy, to develop individualized comprehensive management plans for patients with multiple chronic diseases; (3) establish precise prevention and control mechanisms for key populations such as those with low income and high risk of alcohol dependence, and fully implement comprehensive and integrated management strategies in their co-morbidity screening, intervention plan formulation, and long-term follow-up management, fundamentally avoiding the limitations of single disease management. Through these strategies, promote the liver cirrhosis management model to shift from being mainly based on medical intervention to being centered on community health governance, and comprehensively enhance the public health prevention and control effect of liver cirrhosis.

It should be noted that this study has some limitations. Due to the sampling method and the hierarchical diagnosis and treatment system, the proportion of mild patients from grassroots medical institutions in the sample is relatively low, and the case composition of grassroots patients mainly consists of difficult and critical cases. Therefore, although the sample of this study fully reflects the typical case characteristics of the provincial-level medical treatment center and has good representativeness for similar provincial-level medical institutions, the results should still be interpreted with caution when applied to primary medical institutions. Future research will further verify the application effect and promotion value of this questionnaire by increasing the sample size and conducting multi-center cross-regional surveys.

## 5 Conclusion

The self-health management ability of patients with DLC in Shanxi Province is at a moderately low level and needs to be improved urgently. Education level, occupational status, monthly family income, healthcare burden, duration of liver cirrhosis, alcohol consumption history, comorbidities, and Child-Pugh classification are the key factors affecting their self-health management ability. Based on this, a precise intervention strategy is proposed to enhance the public health prevention and control effect of liver cirrhosis: transitioning from traditional hospital-centric models to a stratified, intelligent, and community-integrated comprehensive prevention and control system.

## Data Availability

The raw data supporting the conclusions of this article will be made available by the authors, without undue reservation.
